# Novel pathogenic *NPR2* variants in short stature patients and the therapeutic response to rhGH

**DOI:** 10.1186/s13023-023-02757-8

**Published:** 2023-07-27

**Authors:** Hong Chen, Suping Zhang, Yunteng Sun, Jiao Chen, Ke Yuan, Ying Zhang, Xiaohong Yang, Xiangquan Lin, Ruimin Chen

**Affiliations:** 1 Endocrinology Department, Fuzhou Children’s Hospital of Fujian Medical University, Fuzhou, 350005 Fujian China; 2 Laboratory Center of Fuzhou Children’s Hospital, Fujian Medical University, Fuzhou, Fujian Province China; 3 Department of Pediatrics, The Lin’an People’s Hospital, Hangzhou, Zhejiang Province China; 4 Department of Pediatrics, The First Affiliated Hospital of Zhejiang University School of Medicine, Hangzhou, Zhejiang Province China

**Keywords:** *NPR2* variants, Short stature, Loss-of-function variants, rhGH treatment

## Abstract

**Objective:**

Heterozygous loss-of-function variants in the *NPR2* gene cause short stature with nonspecific skeletal abnormalities and account for about 2 ~ 6% of idiopathic short stature. This study aimed to analyze and identify pathogenic variants in the *NPR2* gene and explore the therapeutic response to recombinant growth hormone (rhGH).

**Methods:**

*NPR2* was sequenced in three Chinese Han patients with short stature via exome sequencing. In vitro functional experiments, homology modeling and molecular docking analysis of variants were performed to examine putative protein changes and the pathogenicity of the variants.

**Result:**

Three patients received rhGH therapy for two years, and two *NPR2* heterozygous variants were identified in three unrelated cases: c.1579 C > T,p.Leu527Phe in patient 1 and c.2842dupC,p.His948Profs*5 in patient 2. Subsequently, a small gene model was constructed, and transcriptional analysis of the synonymous variant (c.2643G > A) was performed in patient 3, which revealed the deletion of exon 17 and the premature formation of a stop codon (p.His840Gln*). Functional studies showed that both NPR2 variants, His948Profs*5 and His840Gln*, failed to produce cGMP in the homozygous state. Furthermore, the Leu527Phe variant of NPR2 was almost unresponsive to the stimulatory effect of ATP on CNP-dependent guanylyl cyclase activity. This loss of response to ATP has not been previously reported. The average age of patients at the start of treatment was 6.5 ± 1.8 years old, and their height increased by 1.59 ± 0.1 standard deviation score after 2 years of treatment.

**Conclusion:**

In this report, two novel variants in *NPR2* gene were described. Our findings broaden the genotypic spectrum of *NPR2* variants in individuals with short stature and provid insights into the efficacy of rhGH in these patients.

**Supplementary Information:**

The online version contains supplementary material available at 10.1186/s13023-023-02757-8.

## Introduction

Short stature is defined as a body height of two standard deviations score (SDS) below the average of the population (of the same gender and chronological age), and it is one of the most common causes of referral to pediatric endocrinologists [1]. During long bone development, chondrocytes of the cartilaginous growth center, called the epiphyseal growth plate, are responsible for driving linear growth. The regulatory mechanisms of growth plate are complicated and involve multiple factors, such as extracellular matrix molecules, intracellular signaling pathways, paracrine factors, endocrine factors and epigenetic regulation [2]. In recent years, genes involved in paracrine functions, such as C-type natriuretic peptide (CNP) and its receptor atrial natriuretic peptide receptor 2 (NPR2), Indian Hedgehog signaling pathway, parathyroid hormone-related protein, and bone morphogenetic protein/TGF-β superfamily signaling pathway, have attracted more attention, especially NPR2 [3, 4].

CNP and NPR2 are expressed in the hypertrophic region of the growth plate and are major regulators of endochondral ossification [5, 6]. CNP-NPR2 stimulates the production of chondrocytes and cartilage matrix, which promotes the formation of growth plates [7, 8]. Moreover, recent studies have shown that CNP-NPR2 facilitates autonomic Ca^2+^ entry in growth plate chondrocytes for stimulating bone growth [9]. The *NPR2* gene heterozygous loss-of-function variant causes short stature with non-specific skeletal abnormalities (MIM#616,255), accounting for about 2 ~ 6% of idiopathic short stature (ISS) cases [10]. rhGH treatment appears to be beneficial in short-stature patients with *NPR2* heterozygous variants [4]. However, relatively little information is available on short stature due to *NPR2* gene variants. Furthermore, the investigation of genotype-phenotypes and rhGH therapy efficacy correlations requires functional and clinical analysis, including parameters such as age, treatment dose, and duration.

In this study, the clinical features, laboratory results, genetic information, treatment, and follow-up data of three patients with short stature were analyzed. In addition, a series of functional assays and molecular dynamics simulations were performed to elucidate the pathogenic mechanisms associated with *NPR2* variants.

## Patients and methods

### Subjects

This study was approved by the Ethics Committee of Fuzhou Children’s Hospital of Fujian Medical University (approval number 201,921) and was conducted in agreement with the Declaration of Helsinki. Written informed consent was obtained from their parents.

### Clinical evaluations

Medical history and clinical manifestations were assessed in all patients, and physical examinations were performed. Weight and height were measured, and growth hormone stimulation testing was performed using L-dopa (10 mg/kg) and arginine (0.5 g/kg). The levels of serum total T3, total T4, thyrotropin hormone, GH, IGF1 and IGFBP3 were examined by chemiluminescent immunoassay. Bone age was assessed using a radiograph of the left hand and wrist. All radiographs were analyzed by a single endocrinologist who was blinded to the age of the individuals. The Tanner-Whitehouse III (TW3) method adapted for the Chinese population was used to assess bone age [11]. The rhGH therapy was adjusted according to the height increase, IGF-1 levels, and related examination results. The SDS for IGF-1 was calculated from the IGF-1 levels in healthy children and adolescents of the same age and sex [12]. The patients were followed up every three months during rhGH treatment.

### Genetic examination

A standard procedure was used to obtain genomic DNA from the probands and their parents via peripheral blood leukocytes. As described previously, exome sequencing was performed [13]. The *NPR2* gene was described according to the NCBI entry NG_009249 (NM_003995.4), and the nomenclature for sequence variants was adopted from the Human Genome Variation Society (HGVS) [14].

### Conservation and pathogenicity analysis of the variant

Conservation analysis of amino acids was completed as previously described [15]. The PREDICTSNP webserver (https://loschmidt.chemi.muni.cz/predictsnp1/) was used to predict the changes in NPR2 function caused by missense variants. The tool employs a consensus of several different predictors (PredictSNP, PhD-SNP, SIFT, PolyPhen-2, SIFT, and SNAP) along with a confidence score of the predictions [16].

### Minigene construction

The wild-type (WT) human *NPR2* cDNA was obtained from NCBI. To confirm the possible consequences of the splice site variant, an in vitro analysis was performed using a minigene splicing assay based on the pSPL3 exon trapping vector (Invitrogen) [17]. The minigene included the 169-bp exon 15, the 147-bp exon 16, the 124-bp exon 17, the 69-bp exon 18, and the introns of 15, 16, 17 and 18. The minigene sequence was then inserted into the multiple cloning site of the pSPL3 vector (restriction sites: EcoRI and BamHI). The pSPL3-WT plasmid contained the gene of interest. Subsequently, the c.2643G > A variant was introduced into the sequence using PCR with mutagenic primers (5’-CCCATGCAAGTGAGAGCCAT-3’), and the PCR products were ligated using the In-Fusion® Snap Assembly Master Mix (TaKara, Beijing, China).

### Homology modeling and molecular docking of human NPR2

The ATP-binding domain of Human NPR2 was modeled as follows. The wild-type of NPR2 (amino acids:512–783) was modeled on a crystal structure (PDB entry 6JUT). Bioinformatic tools from HOPE (https://www3.cmbi.umcn.nl/hope/) were used to model and predict the effects of the p.Leu527Phe variant on NPR2. The 3D structures of ATP were downloaded from the PubChem database (CID: 5957), which were then energy-minimized using the MMFF94 forcefield implemented in Open Babel (http://openbabel.org/)[18]. AutoDock Vina 1.1.2 software was used for molecular docking [19]. Prior to docking, the water molecules, salt ions, and molecule ligands of the receptor protein were removed by utilizing PyMol 2.5 software [20]. The docking box was then set up to contain the active site. Subsequently, all the processed small molecules and receptor proteins were converted to PDBQT format using ADFRsuite 1.0 [21], and the highest-scoring docked model was chosen. Finally, PyMOL 2.5 was used to visualize the docking results. The interactions between amino acid residues were analyzed using DynaMut (http://biosig.unimelb.edu.au/dynamut/)[22].

### Wild-type and mutant *NPR2* gene expression plasmid construction

Plasmid construction and the corresponding design strategy were described previously [15]. Wild-type full-length human *NPR2* complementary DNA was chemically synthesized and cloned into a 3×FLAG-CMV vector. The c.1579 C > T, c.2842dupC, and c.2643G > A variants were introduced into the *NPR2* sequence using homologous recombination (TaKara, Beijing, China). The ligation mixture was transformed into Escherichia coli DH5α cells. Subsequently, positive clones were PCR-amplified with the universal primers CMV-F (forward) and hGH (reverse), and Sanger sequencing was performed. The mutagenesis primer sequences are listed in Supplementary Table 1.

### Cell culture and transfection

The human embryonic kidney (HEK) 293T cells (ATCC® CRL-11,268) were cultured in high-glucose DMEM (Hyclone®), containing 10% fetal bovine serum and 1% penicillin-streptomycin at 37 °C with 5% CO_2_[15]. HEK293T cells were cultured in 24-well plates. For transient transfections, HEK293T cells at 70% confluence were transfected with 500ng plasmid DNA per well with Hieff Trans™ Liposomal Transfection Reagent (Cat#40,802, Yeasen, China) [15].

### cGMP synthesis and enzyme-linked immunosorbent assay (ELISA)

Seven groups were tested, including a negative control with only plasmid 3×FLAG-CMV, WT with 3×FLAG-CMV-WT-NPR2, and experimental groups with 3xFLAG-CMV-MUT-NPR2 of c.1579 C > T, c.2842dupC, c.2643G > A, WT/c.1579 C > T, WT/c. 2842dupC or WT/c.2643G > A. After 24 h, the cells were starved with opti-MEM for 4 h. The cells were pretreated with 0.1mM 3-isobutyl-1-methylxanthine (GLPBIO, Cat#GC11730, USA) for 15 min and then exposed to 100nM CNP (GLPBIO, Cat#GC43328, USA) and different concentrations of ATP (0 ~ 1000nM) (GLPBIO, Cat#GC35420, USA) for another 15 min. Subsequently, 0.1 M HCl containing 0.4% TritonX-100 was added to stop endogenous phosphodiesterase activity, stabilize the released cGMP, and trigger cell lysis. The samples were centrifuged at 1200×g for 3 min to isolate the supernatants from cell lysates. cGMP levels were detected using the cGMP ELISA detection Kit (GenScript, Cat# L00461, USA), which was performed according to the manufacturer’s instructions. At least three independent experiments were conducted. Data were expressed as mean ± SDS and analyzed using the t-test.

### Western blot analysis

Western blotting was performed as described previously [15]. Antibodies against FLAG-tag (Cat#14,793) were purchased from Cell Signaling Technology (Beverley, MA, USA), and antibodies against α-Tublin (Cat#ab52866) were obtained from Abcam (Shanghai, China). The grayscale was measured by ImageJ software. All experiments were carried out in triplicate, and the data were expressed as the ratio of relative optical density to β-actin levels.

### Quantitative real-time polymerase chain reaction

Total RNA was prepared using Trizol reagent (Invitrogen), and reverse transcription into cDNA was completed with HiScript III RT SuperMix (Vazyme Biotech, Cat#R323) for qPCR. Quantitative real-time PCR was performed as previously described [15], and β-actin (*ACTB* gene) was used as a reference gene for normalization.

### Statistical analysis

Western blotting images were processed using Fiji/Image J software (https://imagej.net/Fiji), while Graph Pad Prism 8 was used for statistical analysis. Statistical significance was analyzed using a t-test. At least three independent experiments were conducted to verify the results, and data were represented as mean ± SDS (n ≥ 3). * indicates P < 0.05, ** indicates P < 0.01 and *** indicates P < 0.001.

## Results

### Clinical evaluations

#### Patient 1

The proband, a 7.6-year-old girl, was admitted to the hospital due to short stature since early childhood. She was delivered by cesarean section at gestational week 40. Her birth weight was 2650 g (3rd percentile), and her birth length was 48 cm (-1.0SDS). On admission, her height and weight were 110.2 cm (-3.06SDS) and 17.5 kg (-0.33SDS), respectively, with a sitting height of 62.2 cm and a sitting height/height ratio of 0.56 (2.35SDS). A spinal X-ray revealed slight lumbar scoliosis (Supplementary Fig. 1A). The bone age (BA) was assessed as 5.3 years using the Tanner-Whitehouse 3 (TW3) method (Supplementary Fig. 1B). Furthermore, the GH stimulation test (levodopa and clonidine) revealed GH levels of 7.9 ng/mL (normal: ≥10ng/mL), and IGF1 levels of 116ng/mL (Table 1). The levels of IGFBP3, thyroid function, plasma calcium, phosphate, and parathyroid hormones were all within normal limits, and pituitary magnetic resonance imaging showed a normal pituitary gland. The heights of the father, mother, and grandmother were 167 cm (-0.93SDS), 146.5 cm (− 2.6SDS), and 150 cm (− 1.96SDS), respectively, and her parents had proportionate bodies.

**Table 1:** Clinical data of patients

Items	Patient 1	Patient 2	Patient 3 [23]	
Gender	Female	Female	Male	
Birth weight (kg/percentile)	2.65 /3rd percentile	2.9/10th percentile	3.0/50th percentile	
Birth height (cm/SDS)	48/-1.0	46/-2.18	50/-0.2	
Age at diagnosis	7.6	4.5	6.0	
Height (cm)	110.2	96.3	103.7	
HSDS	-3.06	-2.60	-3.11	
Weight (kg/SDS)	17.5/-0.33	14/-0.33	16.2/-0.24	
Sitting height	62.2	52.2	ND	
Sitting height/height (ratio/SDS)	0.56/2.35	0.54/2.36	ND	
Bone age (years)	5.3	4.5	4.0	
Blood hormonal characteristics				Normal value
Peak growth hormone	7.9	25.6	7.86	≥ 10ng/mL
IGF1(pg/mL/SDS)	116/-1.45	158/+0.93	185/+1.4	
IGFBP3 (mg/L)	3.05	4.55	3.9	0.7 ~ 10 mg/L

SDS: standard deviation score; HSDS: height standard deviation score; IGF1: insulin-like growth factor-1; IGFBP3: insulin-like growth factor binding protein-3; ND: not done

#### Patient 2

Patient 2 was a 4.5-year-old girl admitted due to short stature since early childhood. Her height and weight were 96.3 cm (-2.6SDS) and 14.0 kg (-0.33SDS), respectively. She was delivered by cesarean section at gestational week 40, with a birth weight of 2900 g (10th percentile) and a birth length of 46 cm (-2.18SDS). Her sitting height was 52.2 cm, with a sitting height/height ratio of 0.54 (2.36SDS). The bone age (BA) was 4.5 years (Supplementary Fig. 1C), and a spinal X-ray revealed slight lumbar scoliosis (Supplementary Fig. 1D). The blood hormone levels are listed in Table 1. The heights of the father and mother were 168 cm (-0.77SDS) and 148 cm (− 2.25SDS), respectively.

#### Patient 3

The characteristics of patient 3, including diagnoses and detailed clinical features, have been described in our previous paper [23]. Briefly, the proband, a 6.0-year-old boy, was admitted to the hospital due to growth delay for over 4 years. His height and weight were 103.7 cm (-3.11SDS) and 16.2 kg (-0.24SDS), respectively. He was delivered by cesarean section at gestational week 38, with a birth weight of 3000 g (50th percentile) and a birth length of 50 cm (-0.2SDS). The blood hormone levels are listed in Table 1. The treatment and follow-up are further described below to provide an explanation of the synonymous variant (*NPR2* c.2643 G > A) carried by the patient and verify its pathogenicity.

#### Treatment and follow-up

**Fig. 1:**
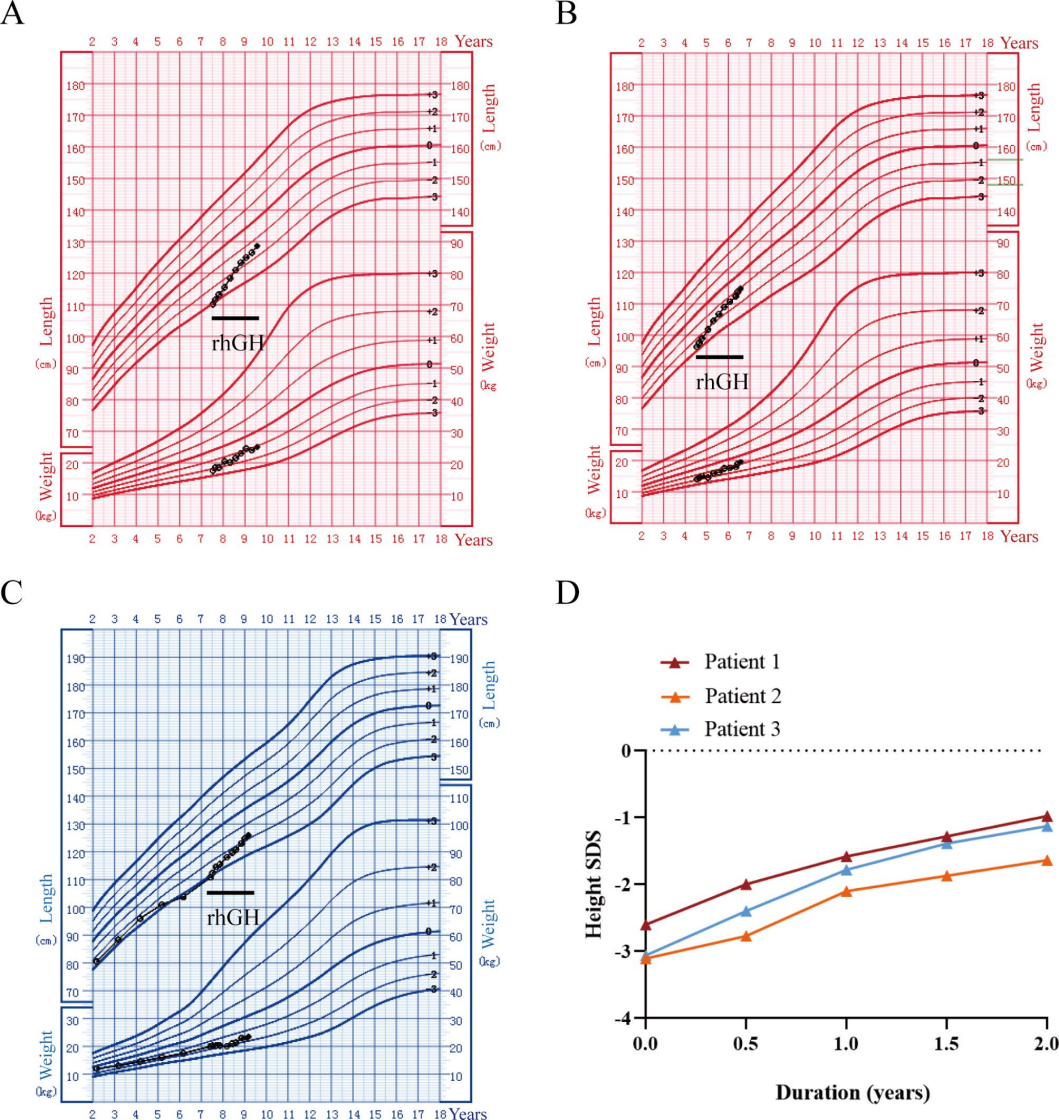
Growth chart of the three patients with heterozygous NPR2 variants with the rhGH therapies. Height and weight standardized growth charts for Chinese children and adolescents aged 0 to 18 years [36]. The black dots represent records. (**A**) Height and weight trends of patient 1 during rhGH treatment. (**B**) Height and weight trends of patient 2 during rhGH treatment. (**C**) Height and weight trends of patient 3 from 2 years old to 9.5 years old. (**D**) rhGH treatment response in the three patients with *NPR2* gene variants

The three patients received rhGH therapy (0.13 ~ 0.15IU/kg/d) for two years (Table 2), with an average treatment initiation age of 6.5 ± 1.8 years. In the first year of treatment, the average height was increased by 1.10 ± 0.15SDS, and the second year of treatment induced an increase in the average height of 0.48 ± 0.11SDS (Fig. 1A and D). The three patients showed a good response to rhGH treatment. No significant side effects were noted during the course of treatment. The details of follow-up, treatment, and treatment outcomes are provided in Table 2.

**Table 2:** Genetic information and response to growth hormone therapy in patients

	Age (years)	Duration of GH treatment (years)	Dose of rhGH (IU/kg/d)	Height (cm)	HSDS	Bone age (years)	IGF1 (pg/mL)	IGFBP3 (mg/L)	Genetic information
Patient 1	7.6	0	0.13	110.2	-3.06	5.8	116	3.05	*NPR2* c.1579 C > T,p.Leu527Phe De novo
	8.1	0.5	0.14	115.5	-2.40	ND	176	3.03	
	8.6	1	0.15	121.0	-1.78	8.0	324	3.45	
	9.1	1.5	0.16	125.0	-1.56	8.6	181	3.74	
	9.6	2	0.17	128.6	-1.40	9.5	153	3.26	
Patient 2	4.5	0	0.15	96.3	-2.60	4.5	158	4.55	*NPR2* c.2842dupC,p.His948Profs*5 Inherited from the mother
	5	0.5	0.15	101.8	-2.00	ND	383	6.58	
	5.5	1	0.14	106.7	-1.58	5.6	318	5.99	
	6	1.5	0.14	110.7	-1.28	ND	236	7.41	
	6.5	2	0.14	114.9	-0.96	6.6	292	6.39	
Patient 3	7.5	0	0.15	110.8	-3.11	4.5	185	3.90	*NPR2* c.2643 G > A, p.His840Gln* Inherited from the father
	8	0.5	0.15	115.8	-2.77	ND	325	5.80	
	8.5	1	0.166	119.7	-2.10	6.6	369	5.94	
	9	1.5	0.17	123.8	-1.87	7.3	334	4.97	
	9.5	2	0.19	128.2	-1.63	8.0	387	5.98	

GH: growth hormone; HSDS: height standard deviation score; rhGH: recombinant human growth hormone; IGF1: insulin-like growth factor-1; IGFBP3: insulin-like growth factor binding protein-3; ND: not done

### Genetic diagnosis

**Fig. 2:**
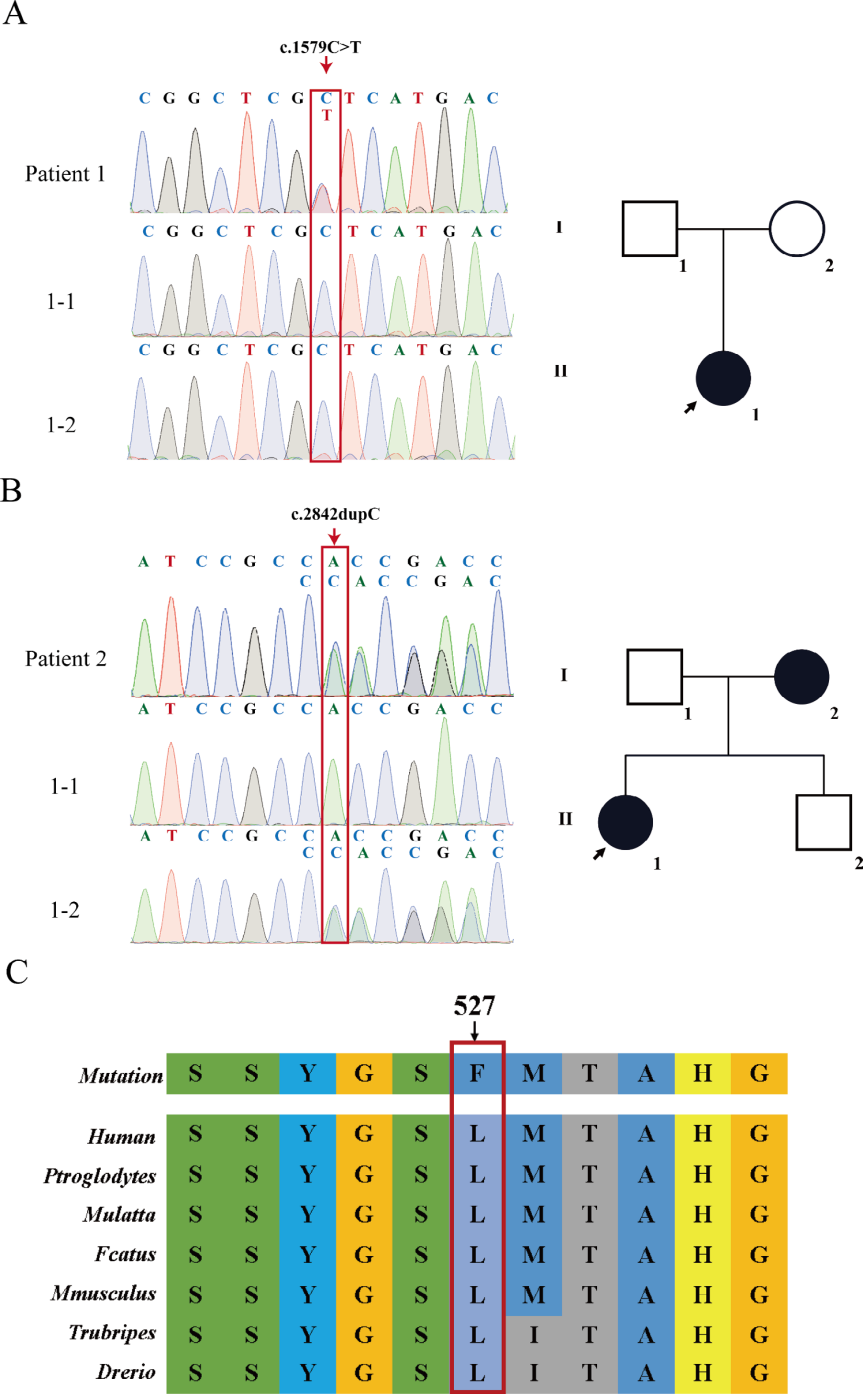
*NPR2* gene variants in the patients and their family members. Squares represent males and circles represent females. The proband is indicated by an arrow. Black-filled symbols indicate subjects with *NPR2* gene variants. (**A**) Sanger sequencing chromatograms show that patient 1 carries the heterozygous c.1579 C > T variant. The parents do not carry the variant. (**B**) Sanger sequencing chromatograms show that patient 2 and her mother (I-2) carry the heterozygous c.2842dupC variant. (**C**) Sequence conservation of mutated amino acids. Arrows point to the positions of Leu527Phe variants

Exome sequencing and Sanger sequencing revealed a heterozygous *NPR2* variant c.1579 C > T,p.Leu527Phe in patient 1 (Fig. 2A). Neither parent carried this variant, suggesting a de novo occurrence. A frameshift variant of the *NPR2* gene (c.2842dupC,p.His948Profs*5) was identified in patient 2, which was inherited from the mother (Fig. 2B). Additionally, neither of the variants was discovered in the publicly accessible gnomAD (https://gnomad.org), Chinese Millionome (https://db.cngb.org), or Human Gene Mutation databases (http://www.hgmd.org/). According to the ACMG/AMP (American College of Medical Genetics and Genomics and the Association for Molecular Pathology) guidelines, the frameshift variant (c.2842dupC) was classified as pathogenic (ACMG criteria: PVS1, PM2 _Supporting, PP1, and PP3), whereas the missense variants appeared as variants of unknown significance (VUS) (c.1579 C > T, ACMG criteria: PM2 _Supporting, PP2, PP3, and PP4). Conservative analysis of the protein sequence showed that Leu527 was highly conserved among various species (Fig. 2D). In addition, four out of five predictors indicated the missense variant Leu527Phe to be deleterious (Table 3).

**Table 3:** Predictions and confidence scores for the NPR2 variants were obtained from the PREDICT-SNP server

Variants Predictors	Leu527Phe	Arg947Pro
PredictSNP (Accuracy)	Deleterious (51%)	Neutral (63%)
PhD-SNP (Confidence scpre)	Deleterious (61%)	Deleterious (73%)
PolyPhen-2 (Confidence scpre)	Deleterious (40%)	Neutral (71%)
SIFT (Confidence scpre)	Deleterious (53%)	Deleterious (43%)
SNAP (Confidence scpre)	Neutral (83%)	Neutral (50%)

**Fig. 3:**
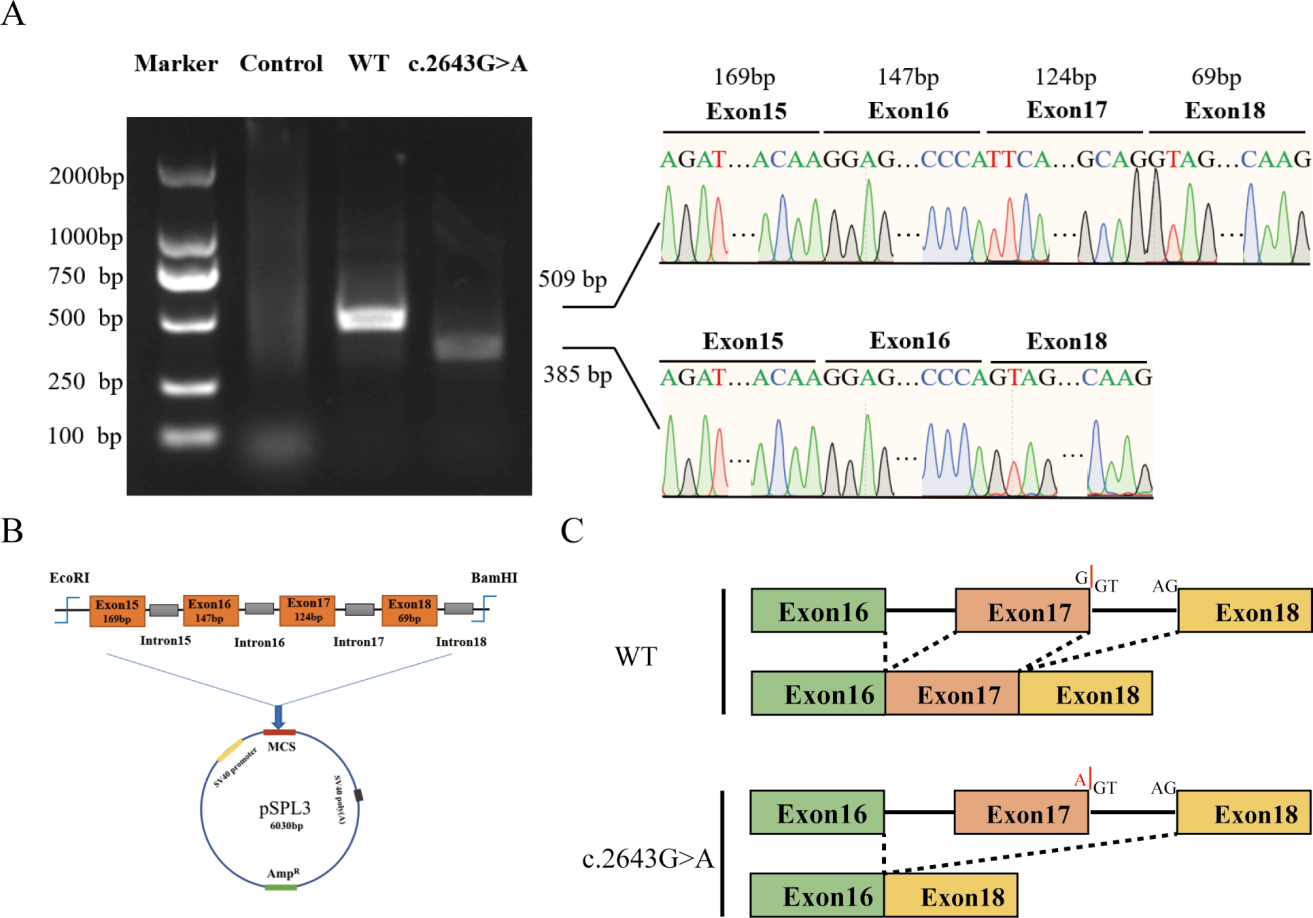
Minigene analysis for NPR2 c.2643G > A. (**A**) Agarose gel electrophoresis of the PCR products. The forward and reverse primers were designed based on the SD and SA sequences and designed for PCR amplification of the cDNA sequence of interest. Lane 1: Marker; Lane 2: empty vector; Lane 3: WT, for which the sequencing of the PCR product revealed that it included the exons SD and SA of pSPL3 and exons 15, 16, 17, and 18 of *NPR2*; and Lane 4: The splice site variant c.2643G > A disrupted the normal splicing, resulting in the deletion of exons 17. (**B**) Schematic overview of the wild-type (WT) minigenes derived from pSPL3. (**C**) Change in splicing of *NPR2* mRNA exons after a G-A mutation

A detailed description of the clinical manifestations and genetic testing results of patient 3 has been reported in our previous study [23]. Briefly, a synonymous variant (*NPR2* c.2643G > A) was identified in patient 3 that appeared to affect mRNA splicing. To assess the effect of the *NPR2* c.2643G > A variant on splicing, a minigene model was constructed and was transiently transfected into HEK293T cells for expression. The empty pSPL3 plasmid vector was used as a negative control, and WT was used as a positive control. Expectedly, a significant difference in the cDNA fragment size was observed between WT and c.2643G > A by agarose gel electrophoresis. WT produced a PCR product of 509 bp, whereas c.2643G > A only produced a 385 bp band, which corresponded to the exons 15, 16, and 18 (Fig. 3A and B). Thus, the c.2643G > A variant caused a complete deletion of exon 17 and a truncation of the NPR2 protein (His840Gln*) (Fig. 3C).

### Wild-type or mutant *NPR2* gene expression in HEK293T cell lines

**Fig. 4:**
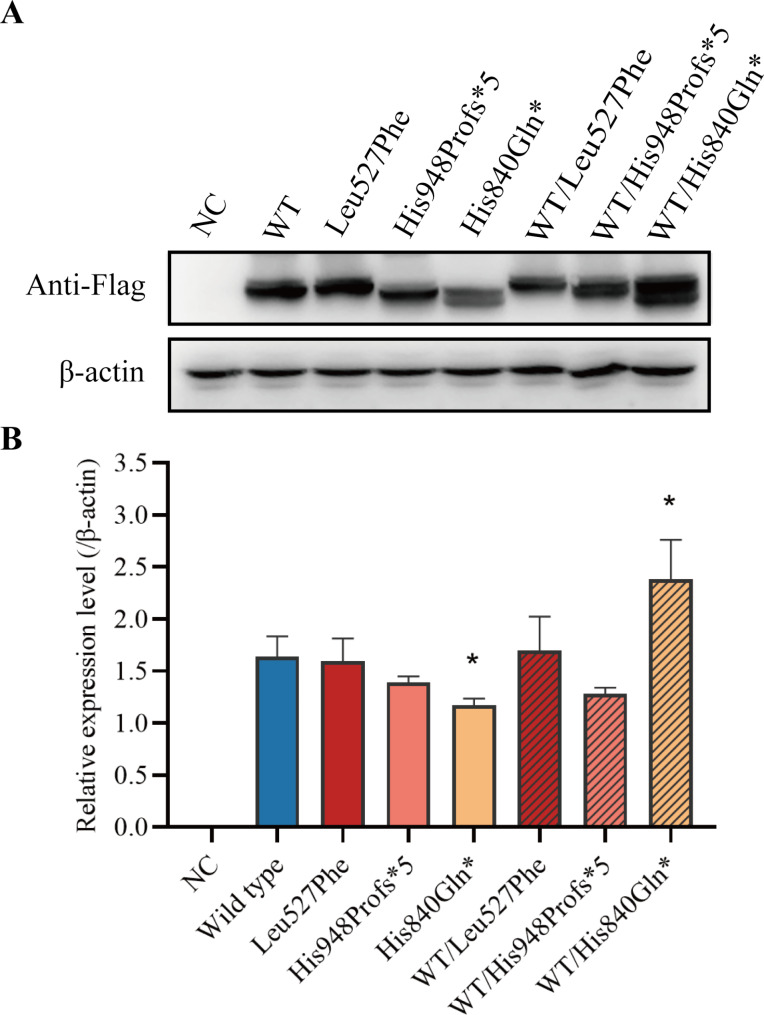
Wild-type or mutant *NPR2* gene expression. HEK293T cells were transfected with wild-type *NPR2* or mutant *NPR2* and were subjected to Western blotting analysis. (**A**) Western blotting analysis of wild-type and variant NPR2 expression. β-actin Western blot is shown below as a loading control. NC: negative control. (**B**) The quantitative estimation of the intensity of bands from the western blot experiment (n = 3); mean ± SD shown; *p < 0.05, ** p < 0.01, ***p < 0.001 by t-test

The wild-type and mutant NPR2 genes were expressed in HEK293T cells to assess the effect of each NPR2 variant. No difference in NPR2 mRNA levels was observed among all of the groups (Supplementary Fig. 2). His948Profs*5 and His840Gln* exhibited a smaller molecular weight than the wild type, consistent with truncated variants. The protein level of His840Gln* was slightly decreased, while the protein expression of other variants showed no significant change. Furthermore, the protein levels after co-transfection of His840Gln* and wild-type was 1.4 times higher than that of the wild-type in HEK293T cells (Fig. 4A and B).

### Functional characterization of the NPR2 variants

**Fig. 5:**
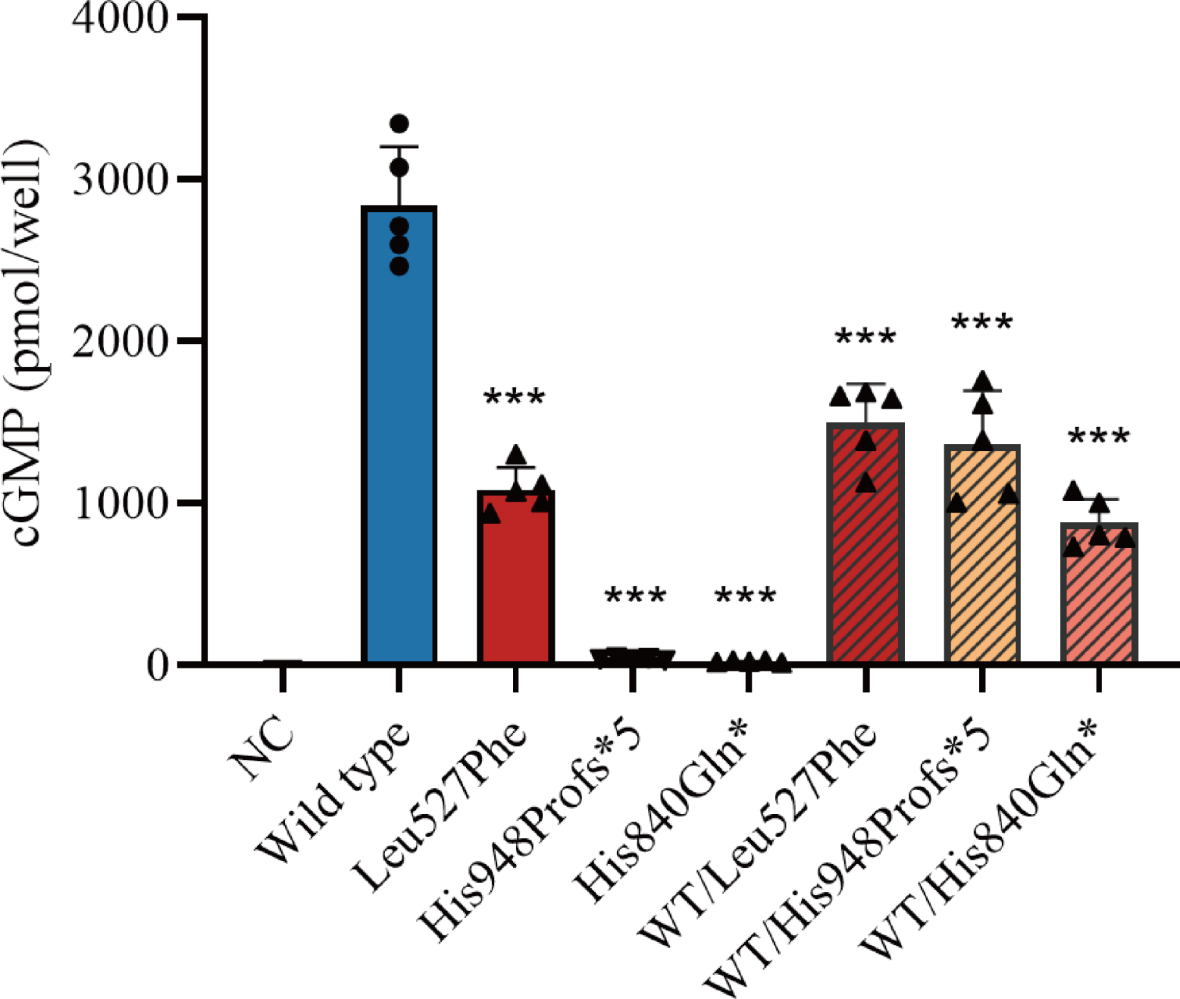
Functional characterization of the NPR2 variants. HEK293T cells were transiently transfected with wild-type or mutant (Leu527Phe) NPR2, or both, and were incubated with CNP (100 µM, 30 min). Whole-cell cGMP contents were quantified by ELISA (n = 5); mean ± SD shown; *p < 0.05, ** p < 0.01, ***p < 0.001 by t-test

The CNP-dependent guanylate cyclase activity of the NPR2 variants was assessed to confirm their predicted pathogenicity. Both NPR2 variants, His948Profs*5 and His840Gln*, failed to produce cGMP in the homozygous state. When co-transfected with the wild-type construct (ratio 1:1), cGMP synthesis showed a significant decrease of 49% and 31% compared to the wild-type. Moreover, the Leu527Phe variant demonstrated a 38.2% cGMP production capacity compared with the wild type, while the heterozygous variant WT/Leu527Phe retained 50.2% of the activity of the wild-type (Fig. 5).

### Homology modeling and molecular dynamics simulation

The protein kinase domain of NPR2 was modeled on the HOPE online server. Molecular docking is used to predict the interactions between small molecules (ligands or substrates) and proteins (enzymes) using molecular modeling techniques [24]. Herein, the Vina 1.1.2 software was used to perform a docking study of ATP and the protein kinase domain of NPR2. The docked conformers were evaluated using the Glide (G) Score (kcal/mol). A negative ΔG represents a positive binding affinity between the ligand and receptor. Generally, a ΔG value less than − 6 kcal/mol is considered to promote binding. The ΔG of this complex was − 9.1 kcal/mol, implying a reasonable binding affinity between ATP and the protein kinase domain of NPR2.

**Fig. 6:**
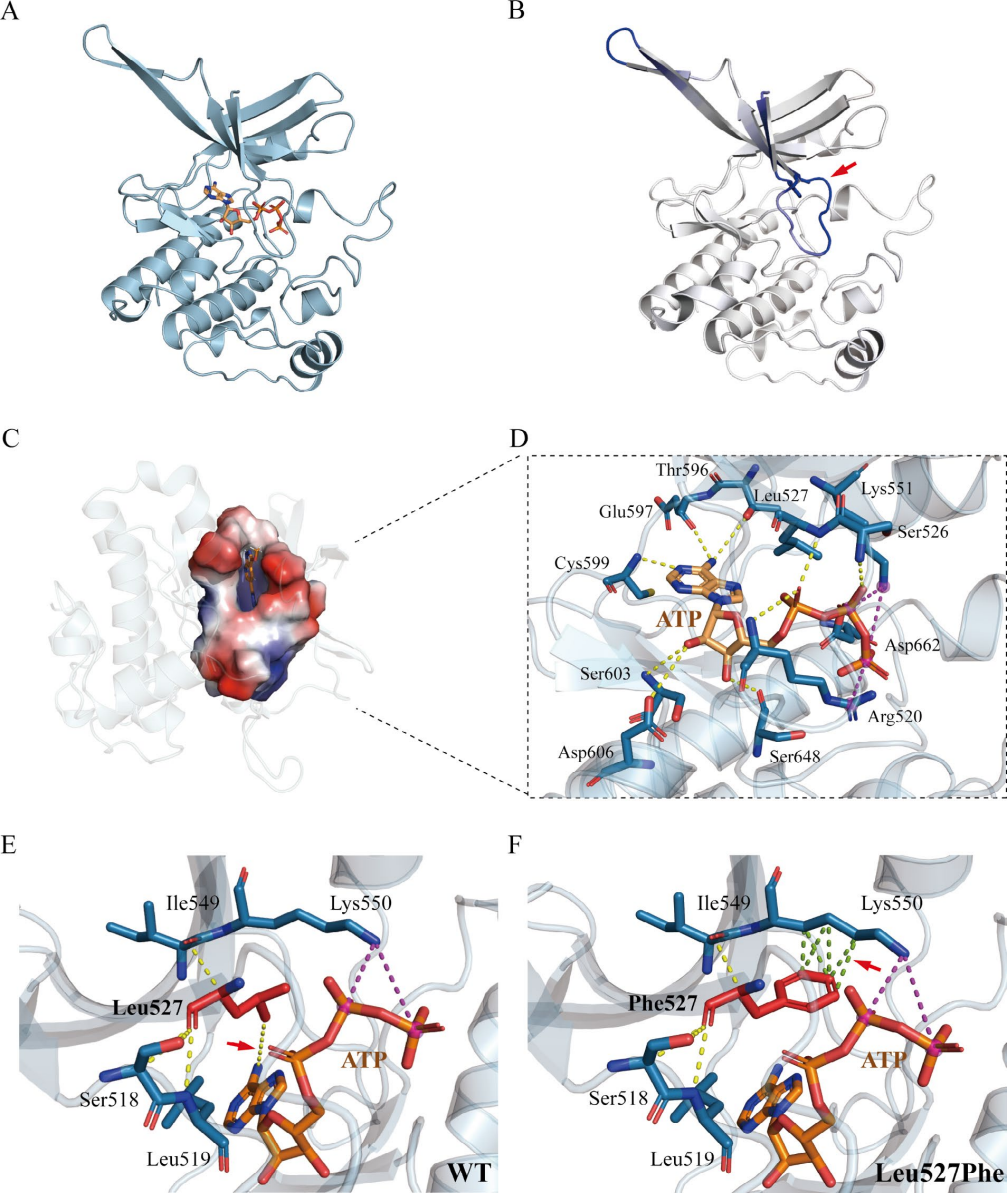
Homology modeling and molecular dynamics simulation. (**A**) The three-dimensional structure was predicted by the HOPE online software. After the removal of the substrate, docking was carried out in standard docking mode. The figure displays an illustration of the protein (blue) and the ATP (stick model in wheat color). (**B**) Δ Vibrational Entropy Energy | Visual representation of variant: Amino acids are colored based on the vibrational entropy change of the variant. Blue represents a rigidification of structure. The red arrows point to a variant at position Leu527Phe. (**C**) The close-up view of the substrate binding pocket of ATP. (**D**) Binding pocket amino acid residue interaction patterns. The yellow dashed lines indicate hydrogen bonds between various amino acids in the binding pockets and ATP. The dotted burgundy line represents the salt bridge interaction. (**D, E**) Interaction prediction between amino acid residues: Wild-type and variant residues are represented as red sticks. These were placed alongside surrounding residues which were also involved in other types of interactions. Red arrows indicate altered intermolecular forces between amino acids. Hydrogen bonding is represented by a yellow dotted line. Hydrophobic bond interactions are represented as green dotted lines

The molecular docking results are displayed in Fig. 6A C. The data showed that the Leu527Phe variant decreased the flexibility of the ATP-binding pocket (ΔΔS_Vib_ENCoM: -0.911 kcal/mol/K) (Fig. 6B). Here, molecular docking results revealed adequate binding between ATP and the active pocket of NPR2. The adenine moiety of ATP formed two hydrogen bonds with the hinge backbone (Glu597 and Cys599) of the kinases (Fig. 6D), and the ribose part of ATP formed hydrogen bonds with Ser603 and Asp606. The phosphate part of ATP also formed hydrogen bonds with Ser648, Arg520, Leu527, and Ser526. In addition, ATP also has salt bridge interactions with Arg520 and Lys551 (Fig. 6D).

Finally, the HOPE and DynaMut online servers were used to predict the local interactions between wild-type amino acid residues and variant amino acid residues. As a result of the variant, leucine (Leu) was changed to phenylalanine (Phe), significantly impacting the height of the individuals. The mutated residue is located in the protein kinase domain, which is important for the binding of ATP. The Leu527Phe variant causes a disruption in the hydrogen bond with ATP, which may impair the binding to ATP. Moreover, the variant introduced a more hydrophobic residue that forms a hydrophobic bond with Lys550, which may disrupt the proper folding of the ATP binding pocket (Fig. 6E F). Thus, the Leu527Phe variant might impair the interaction between ATP and the domain and affect protein function.

### Leu527Phe exchange decreases the ATP-dependent activation of NPR2

**Fig. 7:**
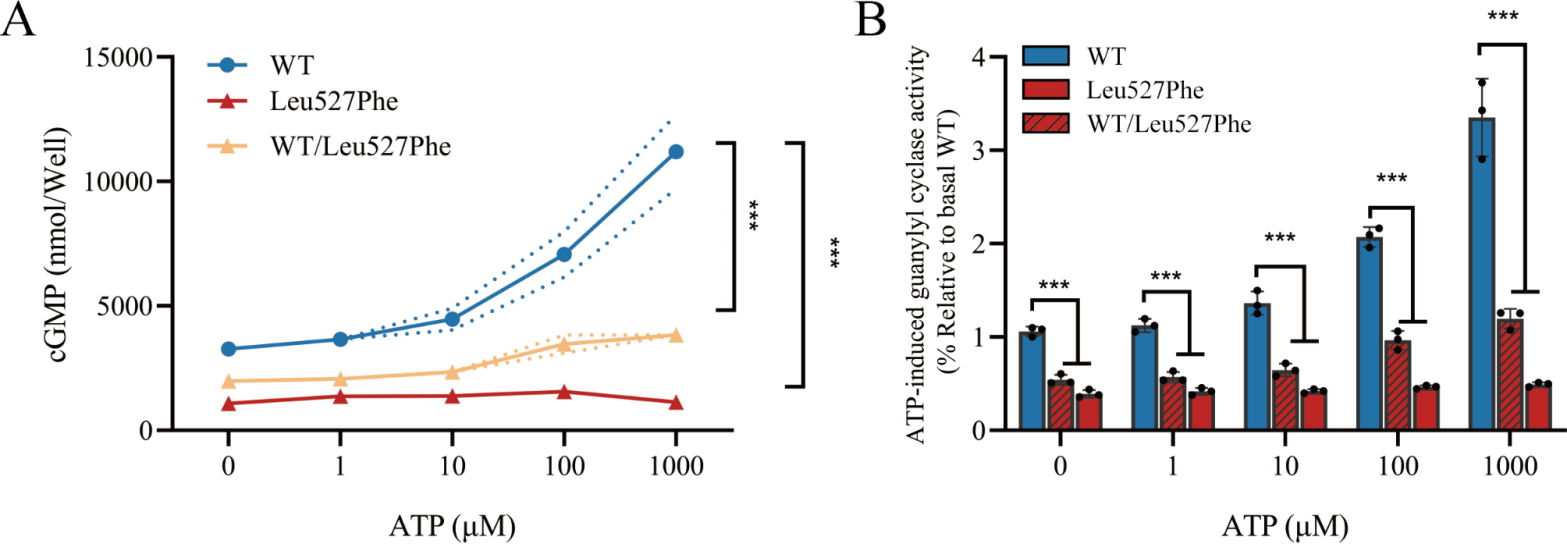
The ATP-dependent activation of Leu527Phe variant. (**A**) Guanylyl cyclase activity was determined in HEK293T cells (expressing wild-type or Leu527Phe variant of NPR2 or both) in the absence or presence of ATP (1–1000 µM) and 100nM CNP (n = 3). The solid line indicates the average, while the dashed line indicates the corresponding standard deviation score. (**B**) The mean wild-type values at each time point were adjusted to 1′, and each black dot represents the relative value of the cGMP level. Each value represents the mean ± SD from at least three independent cultures (***p < 0.001 by t-test)

To investigate the influence of the Leu527Phe variant on the modulatory effect of ATP on NPR2 activity, CNP-induced guanylyl cyclase activity (100nM CNP) assays were performed in the presence of different concentrations of ATP. HEK293T cells were transfected with wild-type or mutant *NPR2* genes (500 ng/well) or cotransfected with the wild-type and mutant *NPR2* genes (ratio 1:1). In wild-type NPR2, significant increases in cGMP levels were observed as the ATP concentration was increased: 1.3-fold (in response to 10 µM ATP), 2.0-fold (100 µM ATP), and 3.2-fold (1000 µM ATP) increase compared to baseline activity. However, the Leu527Phe variant was unresponsive to ATP. When the ATP concentration was increased to 100 and 1000 µM, the cGMP levels of the Leu527Phe variant were approximately 22.3% and 14.4% that of the wild type, respectively. In contrast, the cGMP levels of co-expressed NPR2 were approximately 46.6% (100 µM ATP) and 35.7% (1000 µM ATP) of the wild type (Fig. 7A and B).

## Discussion

The *NPR2* gene has been implicated as a cause of short stature by heterozygous loss-of-function variants [25]. As a homodimeric receptor, NPR2 regulates skeletal growth by activating guanylate cyclase upon binding to its ligand [25, 26]. The NPR2 protein consists of four domains, including the extracellular ligand-binding domain, the transmembrane domain, the kinase homology domain (KHD), and the guanylyl cyclase domain (GCD) [27]. The *NPR2* gene is located on chromosome 9p13.3 and contains 22 exons. Two variants of the *NPR2* gene were identified in patient 1 and patient 2, both of which are novel. Patient 3 carried a synonymous variant (*NPR2* c.2643 G > A) [23] at a conserved site of the intron-exon boundary region. A minigene model was constructed with the exon capture plasmid pSPL3 for transcript analysis. The variant resulted in aberrant splicing, in which exon 17 was skipped, leading to the deletion of exon 17 and the premature formation of a stop codon (*NPR2* gene c.2643G > A,p.His840Gln*). Therefore, this synonymous variant is pathogenic, possibly affecting splicing. Functional studies showed that cells co-transfected with the His840Gln* variant and wild-type NPR2 exhibited a significant decrease in cGMP levels after CNP stimulation in comparison with wild-type, suggesting a dominant-negative effect.

Most ISS-associated NPR2 variants result in abnormal protein trafficking to the plasma membrane, reduced CNP binding affinity for ligands, or inhibition of NPR2 activity [28]. These mechanisms lead to a significant reduction in cGMP production. Studies have shown that ATP binding in the KHD is required for sensitization of NPRs, which is followed by ligand-induced conformational change and enzyme activation [29, 30]. Hannema et al. have reported an activating variant in KHD that significantly increases the stimulatory effect of ATP on CNP-dependent guanylyl cyclase activity, resulting in increased function [27]. The c.1579 C > T variant described in patient 1 resulted in the replacement of leucine with phenylalanine at residue 527 in KHD. As shown in Fig. 6, the modeling of KHD indicated that Leu527 was located in the ATP binding pocket and directly bound to ATP through hydrogen bonding. The prediction results of the DynaMut online server showed a significant change in the local structure of the Leu527Phe variant, and the rigidification of the local protein increased with unique structural disturbance of Leu527Phe, including changes in local hydrogen bond and hydrophobic bond patterns for NPR2. These results suggest that the Leu527Phe variant may impair the interaction with ATP. This hypothesis is supported by the almost unresponsive stimulatory effect of ATP on CNP-dependent guanylyl cyclase activity in the Leu527Phe variant of NPR2.

Heterozygous variants in the *NPR2* gene are associated with short stature, leading to nonspecific clinical manifestations [4, 31, 32]. Patient 1 and patient 2 presented with disproportionate short stature and scoliosis. Recent studies have shown significant differences in the efficacy of rhGH treatment for short stature caused by variants of the *NPR2* gene [4, 31, 33, 34]. Vasque et al. found that GH did not improve height in older children (13 years old) [31]. A recent study showed that rhGH treatment significantly improved height in patients with *NPR2* heterozygous variants and was inversely related to age at treatment initiation [4]. In addition, the effect of growth hormone treatment was also related to the location of the variant. Patients with variants in the extracellular ligand-binding domain typically respond poorly to growth hormones [4]. The children in our study started treatment at a younger age, on average, about 6.5 ± 1.8 years old. Height improved by 1.59 ± 0.1SDS over 2 years of treatment, with a height improvement of 1.10 ± 0.15SDS in the first year. All three patients showed a good response to growth hormone treatment. Due to the progressive development of short stature in patients with *NPR2* variants [35], rhGH treatment is worth considering to prevent short stature. However, the efficacy of rhGH treatment in patients with *NPR2* heterozygous variants still needs to be investigated in a larger population with long-term follow-up.

In conclusion, we report 2 cases of novel pathogenic variants in the *NPR2* gene. The Leu527Phe variant in KHD significantly decreases the stimulatory effect of ATP on CNP-dependent guanylyl cyclase activity, resulting in loss of function. Our results suggest that KHD, especially the ATP-binding structural domain, may participate in regulating the guanylate cyclase activity of NPR2. This study enriches our knowledge of the *NPR2* gene variant spectrum. rhGH therapy may be an effective approach to improving height in patients with heterozygous variants in the *NPR2* gene, particularly those located in the cytosolic compartment.

## Electronic supplementary material

Below is the link to the electronic supplementary material.

Supplementary Material 1

Supplementary Material 2

**Supplementary Table 1**. Mutagenesis primer sequences.

## Data Availability

The dataset supporting the conclusions of this article is included within the article. Novel variants have been submitted to ClinVar (http://www.ncbi.nlm.nih.gov/clinvar/) [Accession numbers: SCV002540213 (*NPR2* c.1579 C > T,p.Leu527Phe) and SCV002498790 (*NPR2*, c.2842dupC,p.His948Profs*5)].

## Abbreviations

ACMG/AMP: American College of Medical Genetics and Genomics and the Association for Molecular Pathology
BA: Bone age
CNP: C-type natriuretic peptide
GCD: Guanylyl cyclase domain
HGVS: Human Genome Variation Society
KHD: Kinase homology domain
NPR2: Natriuretic peptide receptor 2
rhGH: Recombinant growth hormone
SDS: Standard deviations score
WT: Wild type
