# Supplementary figures and images for: Novel pathogenic NPR2 variants in short stature patients and the therapeutic response to rhGH
Source: Orphanet J Rare Dis. 2023 Jul 27;18:221. doi: 10.1186/s13023-023-02757-8 (PMC10375756; doi:10.1186/s13023-023-02757-8)

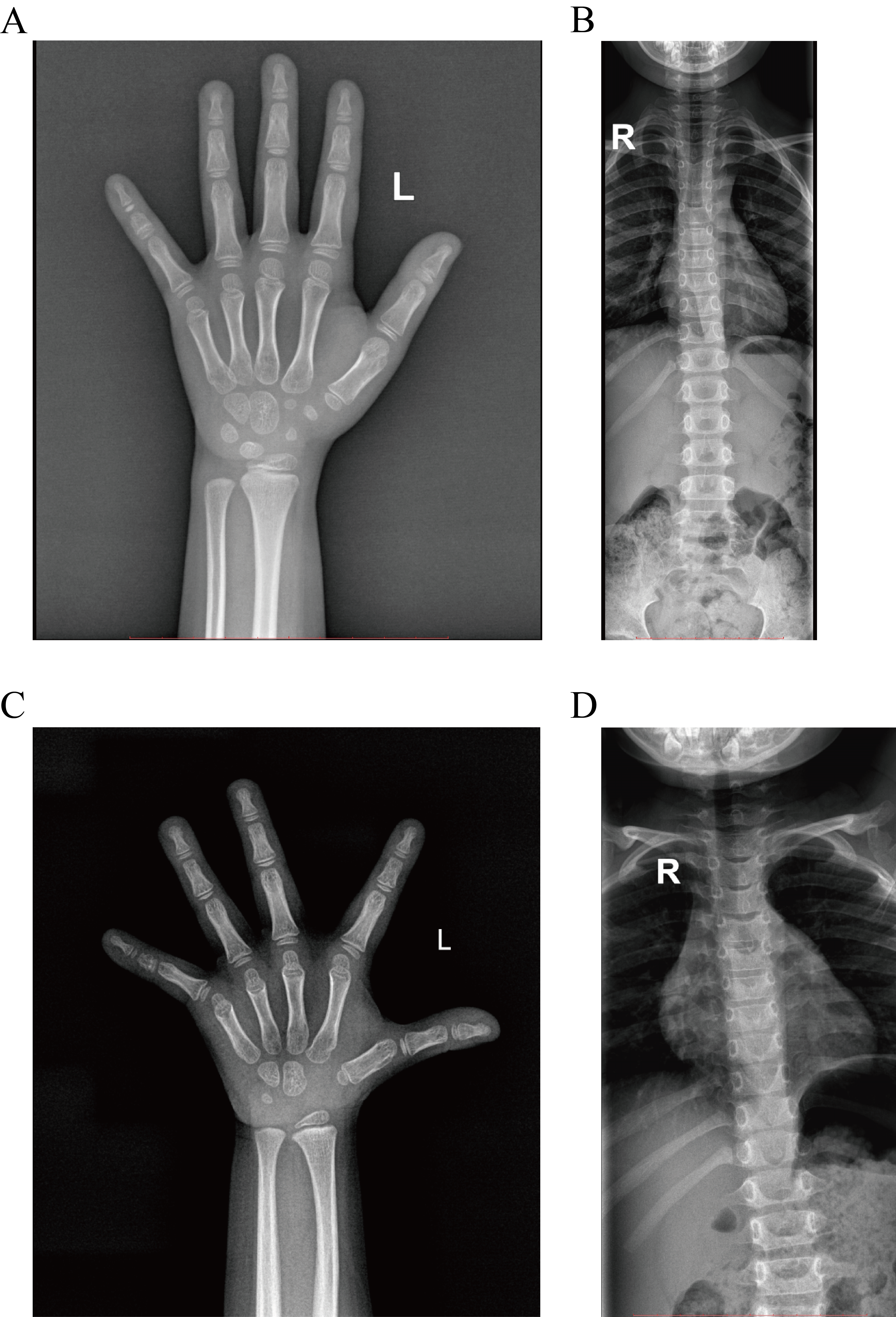

Supplement: Supplementary file 1 — Supplementary Material 1 [file 13023_2023_2757_MOESM1_ESM.png]

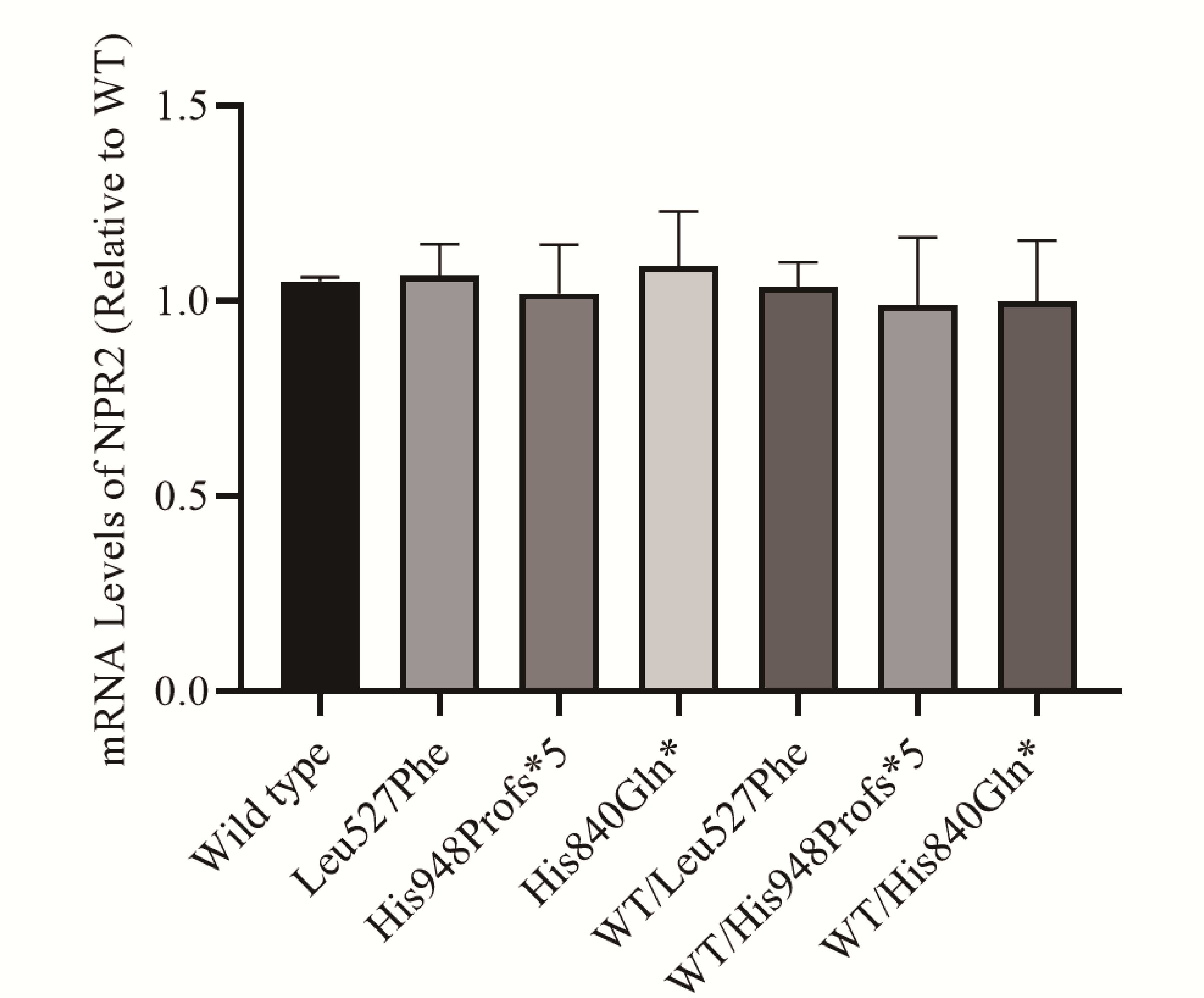

Supplement: Supplementary file 2 — Supplementary Material 2 [file 13023_2023_2757_MOESM2_ESM.png]
