# Supplementary material for: Novel pathogenic NPR2 variants in short stature patients and the therapeutic response to rhGH
Source: Orphanet J Rare Dis. 2023 Jul 27;18:221. doi: 10.1186/s13023-023-02757-8 (PMC10375756; doi:10.1186/s13023-023-02757-8)
Supplement: Supplementary file 3 — Supplementary Table 1. Mutagenesis primer sequences. [file 13023_2023_2757_MOESM3_ESM.docx]

**Supplementary Table 1. Mutagenesis primer sequences.**

| **Mutation site** | **Primer sequences** |
| --- | --- |
| c.1579C>T | GTTACGGCTCGTTCATGACA |
| c.2842dupC | TGGGGTCGGTGGGCGGATGCGAAA |
| c.2643G>A | CTGGGGTAGGATTTGGTAG |
